# ITIH4 in Rheumatoid Arthritis Pathogenesis: Network Pharmacology and Molecular Docking Analysis Identify CXCR4 as a Potential Receptor

**DOI:** 10.3390/pathophysiology31030038

**Published:** 2024-09-20

**Authors:** Lovely Joshi, Debolina Chakraborty, Vijay Kumar, Sagarika Biswas

**Affiliations:** 1 Department of Integrative & Functional Biology, Council of Scientific & Industrial Research (CSIR)-Institute of Genomics and Integrative Biology, Delhi University Campus, Mall Road, Delhi 110007, India; 2 Academy of Scientific & Innovative Research (AcSIR), Ghaziabad 201002, India; 3 Department of Orthopaedics, All India Institute of Medical Science (AIIMS), Ansari Nagar, New Delhi 110029, India

**Keywords:** ITIH4, RA, CXCR4, protein–protein interaction, molecular docking, chemokine signaling, immune response, RA-FLS

## Abstract

Elevated levels of Inter-alpha-trypsin-inhibitor heavy chain 4 (ITIH4) have grabbed attention in rheumatoid arthritis (RA) pathogenesis, though its precise mechanisms remain unexplored. To elucidate these mechanisms, a comprehensive strategy employing network pharmacology and molecular docking was utilized. RA targets were sourced from the DisGeNET Database while interacting targets of ITIH4 were retrieved from the STRING and Literature databases. Venny 2.1 was used to identify overlapping genes, followed by Gene ontology (GO), Kyoto Encyclopedia of Genes and Genomes (KEGG) through Cytoscape 3.10.2 software, and molecular docking was performed in the ClusPro server. The study identified 18 interacting proteins of ITIH4 associated with RA, demonstrating their major involvement in the chemokine signaling pathway by enrichment analysis. Molecular docking of ITIH4 with the 18 proteins revealed that C-X-C chemokine-receptor type 4 (CXCR4), a major protein associated with chemokine signaling, has the highest binding affinity with ITIH4 with energy −1705.7 kcal/mol forming 3 Hydrogen bonds in the active site pocket of ITIH4 with His441, Arg288, Asp443 amino acids. The effect of ITIH4 on CXCR4 was analyzed via knockdown studies in rheumatoid arthritis fibroblast-like synoviocytes (RA-FLS), demonstrating the significant downregulation of CXCR4 protein expression validated by Western blot in RA-FLS. In conclusion, it was speculated that CXCR4 might serve as a potential receptor for ITIH4 to activate the chemokine signaling, exacerbating RA pathogenesis.

## 1. Introduction

Rheumatoid arthritis (RA) is a chronic, systemic, and inflammatory autoimmune disease, primarily affecting joints as a site of infection; characterized by swollen synovial membrane due to the influx of cells inside the synovial membrane [1]. Epidemiological studies have shown a 1% global prevalence of RA with more prevalence in women compared to men in a ratio of 3:1 [2,3]. Disease progression in RA initiates with immune dysregulation, which leads to the generation of autoimmune responses via innate and adaptive mediators [4]. Innate effector cells such as monocytes/macrophages, neutrophils, and mast cells primarily contribute towards the development of inflammation in synovium via pro-inflammatory cytokines such as Tumor necrosis factor alpha (TNF-α), Interleukin-6 (IL-6) production and reactive oxygen intermediates, nitrogen intermediates, production of prostanoids. While cells of the adaptive immune system fuel chronic inflammation via autoantibody production against autoantigens such as post-translational modified proteins (citrullinated, carbamylated) [5]. The major diagnosis parameters are Rheumatoid factor (RF), Anti Citrullinated Protein Antibodies (ACPA), C Reactive Protein (CRP), and Erythrocyte Sedimentation Rate (ESR) that mark disease at certain extents. However, the identification of new disease-specific markers is necessary for the proper diagnosis of disease at an early stage. Moreover, the available treatment includes the use of Disease-modifying anti-rheumatic drugs (DMARDs) and non-steroid Anti-Inflammatory Drugs (NSAIDs), which lack sensitivity and specificity. Therefore, disease-specific drug targets need to be discovered to develop targeted drug delivery for RA remission [6].

A major factor in RA disease progression is Hyaluronic acid (HA), a key component of the extracellular matrix (ECM), which plays pivotal roles in cell signaling, wound repair and regeneration, and matrix organization [7,8]. Increased accumulation of HA has reflected its association with regulating inflammation in RA [9]. Proteomic analysis revealed a significant increase in the levels of the serum-derived hyaluronic acid-associated protein (SHAP-HA) complex in the sera of RA patients [10]. SHAP complex comprises heavy chains of inter-alpha-trypsin inhibitor (ITI) family covalently linked to hyaluronan [11]. The plasma of RA patients showed elevated expression of heavy chain 4, a member of the ITI family [12]. Inter-alpha-trypsin-inhibitor heavy chain 4 (ITIH4) is a 120 kDa protein originally identified in mouse and human plasma, named for its similarity to other ITIH family proteins [13,14]. Predominantly produced and secreted by the liver, its expression increases during acute inflammation. ITIH4, an IL-6-dependent acute phase protein, stabilizes the extracellular matrix by covalently binding with hyaluronic acid to form an active complex [14,15].

Increased expression of ITIH4 was found in plasma and synovial fluid of RA patients [12,16,17]. The citrullinated form of ITIH4 was also found to be differentially expressed in RA patients’ sera and in the joints of both pGIA mice and RA patients and fluctuates with the disease activity score [17]. This suggests that ITIH4 might play a major role in the inflammatory response associated with RA and is a leading factor in disease progression, but its in-depth mechanism remains elusive. Previous in silico studies have generated the 3D model of ITIH4 to understand its functional significance, but the mechanistic role of ITIH4 in RA pathogenesis is still unexplored [18,19].

This study was initiated by screening the interacting proteins of ITIH4, linking them to RA pathogenesis, and further pinpointing specific molecular targets and cellular pathways through molecular docking and pathway enrichment analysis. Further, the in vitro experimentation in rheumatoid arthritis fibroblast-like synoviocytes (RA-FLS) was employed to validate the in silico predictions to understand the mechanistic pathway of ITIH4 through knockdown assay after silencing ITIH4.

## 2. Materials and Methods

### 2.1. Workflow

**Figure 1:**
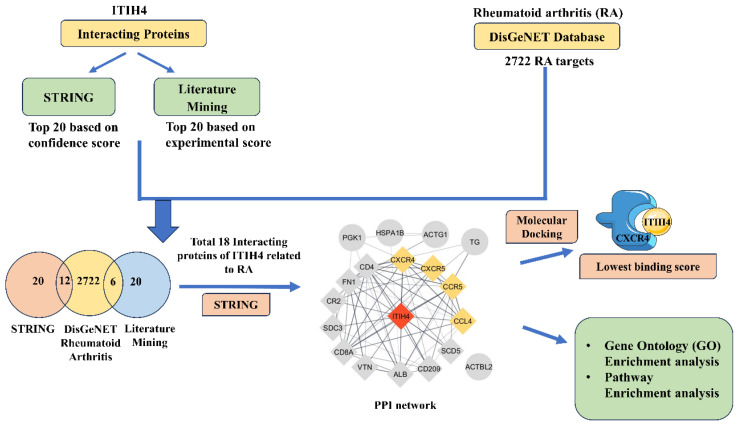
Schematic diagram of the study.

In this study, network pharmacology was used, consisting of three steps. First, overlapping genes interacting with RA and ITIH4 were collected from databases. Second, these overlapping genes were analyzed through enrichment analysis and protein–protein interaction (PPI) networks. Third, the correlation between ITIH4 and RA was further investigated using molecular docking. The research process is illustrated in Figure 1.

### 2.2. In Silico Analysis

#### Screening of Interacting Proteins of ITIH4 and Their Association with RA

The Top 20 interacting genes of ITIH4 were clustered via the STRING database (https://string-db.org/) with medium confidence (0.40 score) [20]. H B Park et al. identified binding partners of ITIH4 by MALDI-TOF/MS analysis and peptide sequence alignment. High scores obtained for the top 20 interacting partners with Sequest HT were also retrieved among them [21].

RA-associated target genes were retrieved from the DisGeNET database, a database of gene-disease associations (https://www.disgenet.org/) [22]. Then, overlapping interacting target genes of ITIH4 for RA were acquired via the Venny 2.1 (https://bioinfogp.cnb.csic.es/tools/venny/ accessed on 5 April 2024) intersection to investigate the relationship between ITIH4 and their potential involvement in the development of RA [23].

### 2.3. Gene Ontology (GO) and Pathway Enrichment Analysis of Common Proteins

Gene ontology (GO) analysis, Kyoto Encyclopedia of Genes and Genomes (KEGG), and Reactome pathway analysis are important methods that describe the associated features and pathway links of candidate targets/proteins. This analysis was performed in Cytoscape 3.10.2 [24]. The GO analysis was applied for target protein analysis, and the top 10 functional categories in biological process (BP), cellular component (CC), and molecular function (MF) were chosen. KEGG and Reactome pathway enrichment analysis was carried out, and analysis was represented by GraphPad 9.0 [25].

### 2.4. Protein–Protein Interaction (PPI) Network Construction of Common Proteins

A PPI network map was constructed for the co-expression, fusion, neighborhood, and co-localization of common proteins with predicted protein interactions. The name of the common proteins was entered via the STRING database, with the “Homo sapiens” category being selected. Each node represents a protein in the PPI network map, and each edge represents a functional association between potential target genes. These results were imported into Cytoscape 3.10.2 [24], and a further PPI network was constructed according to the confidence degree of targeted proteins and their related RA-linked pathways.

### 2.5. Preparation of Ligand Protein for Molecular Docking

The protein structure of ITIH4 was retrieved from the 3D model of the previous study in the .pdb format [19]. The structure was visualized using PyMOL 3.0 software and edited by removing water molecules [26].

### 2.6. Preparation of Receptor Protein

The structure of common proteins: Phosphoglycerate kinase 1 (PGK1) [PDB ID: 3C39], Thyroglobulin (TG) [PDB ID: 6SCJ], Serum Albumin (ALB) [PDB ID: 6M4R], Heat shock 70 kDa protein 1B (HSPA1B) [PDB ID: 7F4Z], Beta-actin-like protein 2 (ACTBL2) [Alpha Fold Structure: AF-Q562R1-F1; UniProt ID: Q562R1], Actin, cytoplasmic 2 (ACTG1) [PDB ID: 6CXI], T-cell surface glycoprotein CD4 (CD4) [PDB ID: 1WIO], CD209 antigen (CD209) [PDB ID: 1K9I], Fibronectin (FN1) [PDB ID: 3M7P], Vitronectin (VTN) [PDB ID: 3BT1], C-X-C chemokine receptor type 4 (CXCR4) [PDB ID: 3ODU], C-C motif chemokine 4 (CCL4) [PDB ID: 2X6L], C-X-C chemokine receptor type 5 (CXCR5) [Alpha Fold Structure: AF-P32302-F1; UniProt ID: P32302], C-C chemokine receptor type 5 (CCR5) [PDB ID: 4MBS], Syndecan-3 (SDC3) [Alpha Fold Structure: AF-O75056-F1; UniProt ID: O75056], Stearoyl-CoA desaturase 5 (SCD5) [Alpha Fold Structure: AF-Q86SK9-F1; UniProt ID: Q86SK9], T-cell surface glycoprotein CD8 alpha chain (CD8A) [PDB ID: 1CD8], and Complement receptor type 2 (CR2) [PDB ID: 3OED]. These were retrieved from PDB and AlphaFold Protein Structure Database [27,28]. These protein preparations were carried out by optimizing protein model geometry by removing water and impurities using PyMOL to ensure accuracy due to the presence of ions, extra molecules, and heteroatoms.

### 2.7. Active Binding Site Prediction

The active site of the ITIH4 structure was predicted by using the Computed Atlas of Surface Topography of Proteins (CASTp 3.0) at http://sts.bioe.uic.edu/castp/ accessed on 9 April 2024. The binding pockets of the ITIH4 structure were visualized along with the amino acids present in the pockets [29].

### 2.8. Molecular Docking

The interaction of ITIH4 with common proteins was conducted by molecular docking using a protein–protein docking software, ClusPro 2.0 server [30]. An energy score was obtained that served as a quantitative measure of their interaction strength [31]. To analyze the binding sites of the protein–protein interactions, the PDBsum database was used [32]. Obtained interactions were represented by LigPlot^+^ v.2.2, a software that automatically creates two-dimensional schematic representations of binding sites involved in protein interactions [33].

### 2.9. Cell Culture and Treatment

#### Collection and Isolation of Primary Cells from Biopsy Synovium

Synovial tissue samples were obtained from RA patients (according to the 2010 ACR/EULAR criteria) (*n* = 3) [34]. Clinical demography and details of the patients are mentioned in Table 1. A signed consent was obtained from each participant, and all the participants were informed about the purpose of the study. The study protocol was ethically approved by the All India Institute of Medical Sciences, New Delhi, India (Reg No IEC-237/07.05.2021, RP-18/2021). All study protocols were compiled with the Declaration of Helsinki.

RA biopsy synovium (~20 mg) was washed with PBS, finely chopped, treated with collagenase (0.5 mg/gm), and incubated for 12–18 h in 30 mL complete media. The undigested tissue was passed through the cell strainer (100 µm pore size, BD), centrifuged at 500× *g* for 5 min, and cultured in a T-75 tissue culture flask in complete Dulbecco Modified Eagle Medium (DMEM) supplemented with 10% Fetal Bovine Serum (FBS) at 37 °C under a humidified atmosphere containing 5% CO_2_. Rheumatoid arthritis fibroblast-like synoviocytes (RA-FLS) were used in the 3rd to 5th passages [35].

### 2.10. In Vitro Knockdown of ITIH4

RA-FLS were cultured and transfected at 60% confluency. The knockdown efficiency of ITIH4 was standardized by transfection with ITIH4 siRNA (Santa Cruz, CA, USA) (25 nM and 50 nM) using Lipofectamine (4 µL/mL) RNAiMAX Transfection Reagent (Invitrogen, Waltham, MA, USA) in Opti-MEM media [35]. Media were replaced after 6 h with complete DMEM media, and cells were harvested after 72 h of transfection [36]. Untreated cells and cells transfected with non-specific siRNA (Santa Cruz, CA, USA) were used as controls.

### 2.11. Western Blot

The cells were washed with PBS, lysed with radio-immunoprecipitation assay lysis buffer (RIPA) (Thermo, Waltham, MA, USA) containing a 1% (*v*/*v*) protease and phosphatase inhibitor cocktail (G-BIOSCIENCES, St. Louis, MO, USA), and incubated (1 h, 4 °C). The lysate was centrifuged (15,000× *g*, 4 °C, 30 min), and 40 µg protein estimated by bicinchoninic acid assay (BCA) was run on 10% sodium dodecyl sulfate–polyacrylamide gel electrophoresis (SDS-PAGE) and transferred to the nitrocellulose (NC) membrane via the wet transfer unit (Bio-Rad, Hercules, CA, USA). The membrane was then incubated (3 h, room temperature) with 3% Bovine Serum Albumin (BSA) followed by incubation (overnight at 4 °C) separately with diluted (1:3000 each) primary antibodies: Anti-ITIH4 (Santa Cruz, CA, USA), Anti-CXCR4 (Cloud clone, Katy, TX, USA), and Anti-β-actin (Santa Cruz, CA, USA). The membrane was washed and incubated (1 h, room temperature) again with horseradish peroxidase (HRP) conjugated anti-mouse secondary antibody (1:6000), developed with an enhanced chemiluminescence reagent (Cyanogen, USA), obtained image by ChemiDoc MP (Bio-Rad, Hercules, CA, USA) and analyzed by Image Lab 5.1 software (Bio-Rad, Hercules, CA, USA) [37].

### 2.12. RNA Isolation and Quantitative Real-Time Polymerase Chain Reaction (qRT-PCR)

After treatment, cells were lysed with 500 µL Tri-Xtract (G-BIOSCIENCES, St. Louis, MO, USA), incubated (5 min, room temperature), added chloroform (200 µL), and centrifuged (15 min, 11,000× *g*, 4 °C). Supernatant was separated, mixed with chilled isopropanol, and incubated again on ice (10 min), followed by centrifugation (15 min, 11,000× *g*, 4 °C). RNA pellet was washed and dissolved in nuclease-free water, and concentration was measured by NanoDrop (Thermo, Waltham, MA, USA). Complementary DNA (cDNA) was then synthesized by using a kit (G-BIOSCIENCES, St. Louis, MO, USA) and used for amplification of ITIH4 using respective primer and HOT FIREPol EvaGreen qPCR Master mix (Solis Biodyne, Tartu, Estonia). The polymerase chain reaction (PCR) was run for 40 cycles using a light cycler-480 system (Roche, Indianapolis, IN, USA) and analyzed by Δct methods. The primers sequences are as follows: ITIH4 (5′-TCTCATCCCGATTTGCCCAC-3′, 5′-TCTCCTTGATGATCCCTGGGT-3′), GAPDH (5′-GAAGGTGAAGGTCGGAGTC-3′, 5′-GAAGATGGTGATGGGA TTTC-3′) [38].

### 2.13. Statistical Analysis

All non-parametric Mann–Whitney tests were performed using GraphPad Prism 9.0. Statistical analysis was performed using the paired Student’s *t*-test to compare the data between two groups, and one-way analysis of variance (ANOVA) was used to compare data among multiple groups. The obtained *p*-values were represented by asterisks on the graph (* *p* ≤ 0.05, ** *p* ≤ 0.01, *** *p* ≤ 0.001, **** *p* ≤ 0.0001).

## 3. Results

### 3.1. Retrieval of Overlapping Targets of ITIH4 Associated with RA

**Figure 2:**
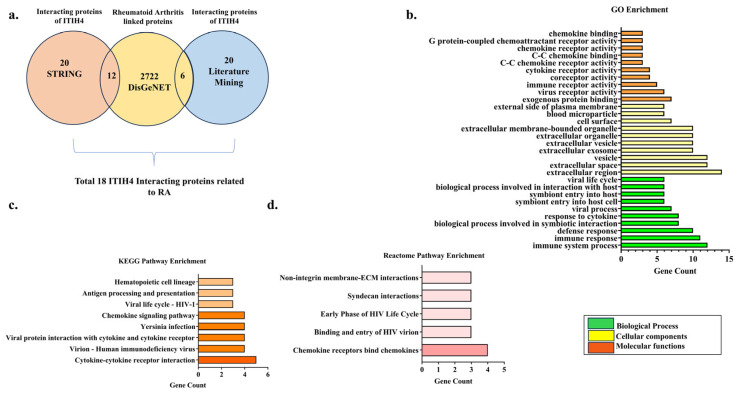
(**a**) Venn diagram representing interacting common proteins of ITIH4 related to RA genes. (**b**) Gene ontology of common ITIH4 interacting proteins. (**c**) KEGG and (**d**) Reactome pathway enrichment of common ITIH4 interacting proteins.

The association of ITIH4 in RA pathophysiology was further explored by retrieval of interacting targets of ITIH4 (Top 20) using the STRING and literature databases. A total of 2722 RA-associated genes were retrieved from the DisGeNET database. Among them, a total of 18 RA targets were found to be shared with interacting targets of ITIH4 (Figure 2a).

The overlapping interacting proteins of ITIH4 were ALB, TG, HSPA1B, PGK1, ACTBL2, ACTG1, VTN, FN1, CXCR4, CXCR5, CCR5, CCL4, SDC3, SCD5, CR2, CD8A, CD4, and CD209, which were found to have a link with RA. This suggests that ITIH4, along with its interacting proteins, might contribute to the progression of RA.

### 3.2. Gene Ontology (GO) Enrichment Analysis

The biological properties of the 18 common targets, including their biological processes, cellular components, and molecular functions, were elucidated by Cytoscape 3.10.2 to illustrate the association of the proteins with RA and its pathogenesis. This illustrated 55 GO entries, out of which 25 entries are associated with biological processes (BP), 15 with Cellular components (CC), and 15 with Molecular functions (MF). The top 10 entries with *p*-values ≤ 0.05 are shown (Figure 2b). The top 10 BP with the highest targets include the immune system process, immune response, defense response, the biological process involved in symbiotic interaction, response to cytokine, viral process, symbiont entry into the host–cell, symbiont entry into the host, the biological process involved in interaction with host and viral life cycle. The top 10 CC with the highest targets include the extracellular region, extracellular space, vesicle, extracellular exosome, extracellular vesicle, extracellular organelle, extracellular membrane-bounded organelle, cell surface, blood microparticle, and external side of the plasma membrane. The top 10 MF with the highest targets include exogenous protein binding, virus receptor activity, immune receptor activity, coreceptor activity, cytokine receptor activity, C-C chemokine receptor activity, C-C chemokine binding, chemokine receptor activity, G protein-coupled chemoattractant receptor activity, and chemokine binding (Supplementary File S1).

### 3.3. Pathway Enrichment Analysis

A target pathway network was created using Cytoscape 3.10.2 to explore the connections between the target proteins and the relevant biological pathways. KEGG pathway enrichment of these proteins derived eight related pathways associated with Cytokine–cytokine receptor interaction, Virion–Human immunodeficiency virus, Viral protein interaction with cytokines and cytokine receptors, Yersinia infection, Chemokine signaling pathway, Viral life cycle—HIV-1, Antigen processing, and presentation and Hematopoietic cell lineage (Figure 2c). We also performed a Reactome pathway analysis of these proteins to enrich more pathways and to deduce their association with RA. The analysis majorly showed the pathways related to chemokine receptors binding chemokines, binding and entry of HIV virion, the early phase of the HIV Life Cycle, syndecan interactions, and non-integrin membrane-ECM interactions (Figure 2d). Overall, the in silico enrichment analysis showed that ITIH4 might be related to chemokine-related signaling involved in rheumatoid arthritis pathogenesis (Supplementary File S1).

### 3.4. PPI Network of the Common Interacting Proteins

**Figure 3:**
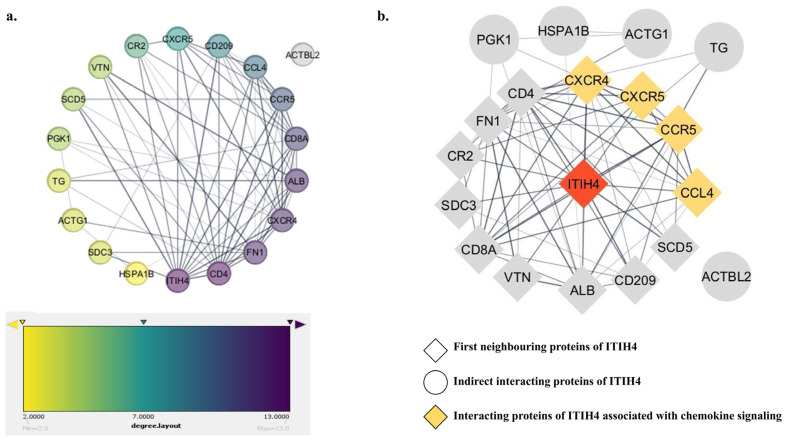
(**a**) PPI of common ITIH4-interacting targets evaluated by STRING. Established by Cytoscape; the node color positively correlates with the degree of contribution in the network. (**b**) PPI network of common proteins highlighting the first neighbors of ITIH4 and proteins associated with Chemokine signaling.

A PPI network was constructed to elucidate the relationships among the 18 common targets. The color of each node reflects its degree of contribution within the network (Figure 3a). The color of nodes like ITIH4, CD4, FN1, CXCR4, ALB, and CD8A, with their degree values of 13, 13, 12, 12, 12, and 11, respectively, was comparatively darker, indicating higher interacting networks with the associated proteins. Hence, these targets could play a direct or indirect role in the advancement of RA.

Furthermore, the first neighbors of ITIH4 were explored, which revealed 13 targets including CXCR4, CCR5, FN1, CD4, CR2, CCL4, VTN, ALB, CD209, SCD5, CXCR5, CD8A, and SDC3 (Figure 3b); nodes are represented in diamond shape. This suggested that most of the interacting targets of ITIH4 are directly linked with RA pathogenesis. The proteins that are involved in chemokine signaling are CXCR4, CXCR5, CCR5, and CCL4 depicted in yellow color nodes.

### 3.5. Predicted Binding Sites of ITIH4

**Figure 4:**
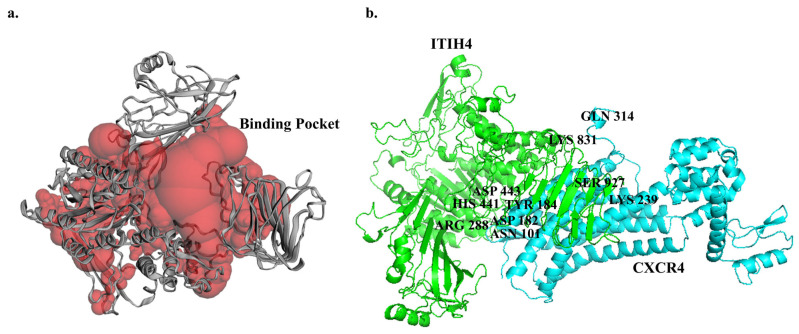
(**a**) Predicted binding pocket (red) of ITIH4 protein using CASTp server. (**b**) Molecular docking of ITIH4 and CXCR4 shown using PyMOL (ITIH4 in green and CXCR4 in cyan).

The active binding pocket of the ITIH4 protein structure was interpreted by the Computer Atlas of Surface Topography of Proteins (CASTp 3.0) server, which depicted a binding volume of 20,563.187 Å3 and surface area of 11,551.837 Å2 (Figure 4a). The binding sites located within the pocket are listed in Table 2 and were subsequently evaluated for docking analysis.

### 3.6. Molecular Docking of ITIH4 with Common Proteins

The interaction or affinity of selected proteins with ITIH4 was analyzed by the in silico protein–protein docking approach using ClusPro 2.0 software. The PyMol software was employed to render the most favorable docked complexes. Among them, M00 emerged as the superior model, determined by cluster size and weightage scores in the protein–protein docking interaction [39]. The strength of binding to the target protein increases as the binding energy value decreases. Among all docking interactions, the best weightage energy score was found to be −1705.7 kcal/mol for the interaction of CXCR4 with ITIH4 (Table 3). The B chain of CXCR4 was found to interact with the single chain of ITIH4 (Figure 4b).

**Figure 5:**
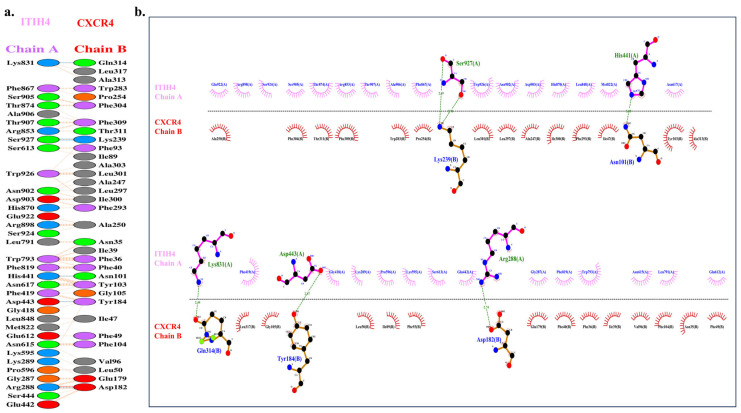
(**a**) H-Bond representation between amino acid residues of ITIH4 and CXCR4. (**b**) Ligplot+ representation of the interaction of ITIH4 and CXCR4. Residues represented in Ligplot+ are labeled, and hydrogen bonds (H-bonds) are represented by dashed lines in Ligplot.

A total of six H-bonds interactions were observed between ITIH4 and CXCR4 (Figure 5a) (Table 4). The residues of ITIH4 involved in the H-bonding were HIS 441: ASN 101; Bond length—3.05, ARG 288: ASP 182; Bond length—2.74, ASP 443: TYR 184; Bond length—2.87, SER 927: LYS 239; Bond length—2.69, SER 927: LYS 239; Bond length—2.70, LYS 831: GLN 314; Bond length—2.44 (Figure 5b). Among them, three residues of ITIH4 (HIS 441, ARG 288, ASP443) were observed to reside in the active binding pocket of ITIH4 protein, indicating a strong interaction of CXCR4 and ITIH4.

### 3.7. ITIH4 Knockdown Reduces CXCR4 Expression in RA-FLS

**Figure 6:**
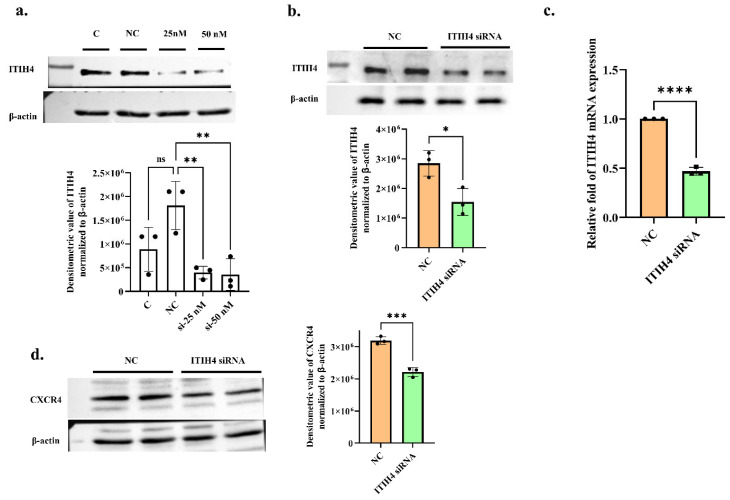
Knockdown of ITIH4 expression in RA-FLS and its effect on CXCR4 level. (**a**) RA-FLS were transfected with NC siRNA or 25 or 50 nM ITIH4 siRNA for 72 h, and then the knockdown level of ITIH4 was investigated through Western blot analysis (*n* = 3). (**b**) ITIH4 protein expression by Western blot was analyzede after ITIH4 silencing at 50 nM in RA-FLS (*n* = 3). (**c**) ITIH4 mRNA expression by qRT-PCR was analyzed after ITIH4 silencing at 50 nM in RA-FLS (*n* = 3). (**d**) Downregulated expression of CXCR4 after ITIH4 knockdown in RA-FLS (*n* = 3). (RA-FLS, Rheumatoid arthritis fibroblast-like synoviocytes; NC, non-specific siRNA; si-ITIH4, small interfering RNA targeting ITIH4; Quantitative Real-Time Polymerase Chain Reaction (qRT-PCR); ns, not significant; * *p* < 0.05; ** *p* < 0.01; *** *p* < 0.001; **** *p* < 0.0001).

To validate the effect of ITIH4 on CXCR4 expression in RA-FLS, the knockdown of ITIH4 was conducted in cells in a dose-dependent manner. The 50 nM si-ITIH4 dose on RA-FLS was able to obtain a knockdown of 70% protein level while the 25 nM dose was seen to knockdown up to ≈65% on protein level compared to the negative control (Figure 6a) with significant *p*-value (*p* ≤ 0.01). ITIH4 expression was decreased at the protein (fold change—0.54, *p* ≤ 0.05) and mRNA levels (fold change—0.46, *p* ≤ 0.0001) at a 50 nM dose of ITIH4 siRNA (Figure 6b,c). CXCR4 protein expression was also found to be significantly downregulated (Fold change—0.69, *p* ≤ 0.001) after ITIH4 knockdown (Figure 6d). This reduction in CXCR4 protein expression after silencing the ITIH4 expression in cells indicates that a decrease in ITIH4 leads to significant CXCR4 suppression in RA-FLS.

**Figure 7:**
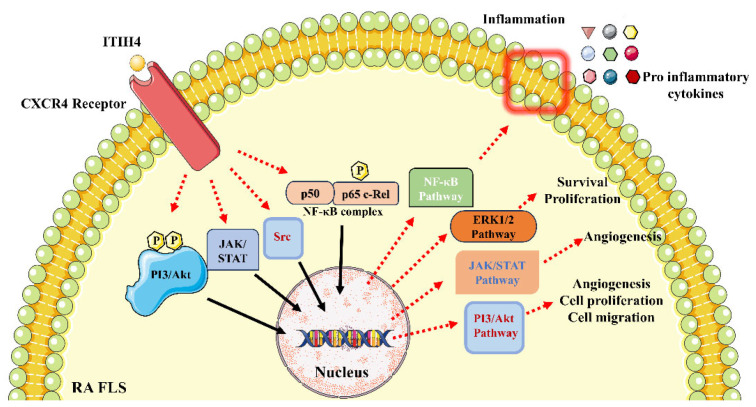
Hypothetical representation of ITIH4 mechanistic pathway via CXCR4 activation in RA synoviocytes.

This in vitro validation indicates that ITIH4 affects CXCR4 expression in RA-FLS. It might become involved in chemokine signaling through CXCR4 in RA-FLS and lead to activating downstream signaling. Earlier studies in the literature reported that CXCR4 signaling pathway activation regulates disease-promoting molecules such as the phosphorylation of the PI3/Akt complex, activation of JAK and Src kinases, phosphorylation of the p65 complex, and their associated pathways including the PI3/Akt/mTOR pathway, the JAK/STAT pathway, and the Ras/Raf/MEK/ERK and NF-κB pathways [40]. Hence, we hypothesize that the binding of ITIH4 with CXCR4 can modulate all the above-mentioned pathways that are major mediators of RA progression (Figure 7).

## 4. Discussion

Rheumatoid arthritis (RA) is an autoimmune disease characterized by the production of autoantibodies, which trigger inflammatory processes involving the production of cytokines and chemokines. This cascade of immune responses leads to chronic synovial inflammation, resulting in synovial hyperplasia and swelling [41]. Over time, the persistent inflammation causes significant cartilage and bone destruction, ultimately leading to joint deformity and loss of function. The involvement of these immune mediators not only sustains the inflammatory state but also promotes the progressive damage observed in RA, highlighting the complexity and severity of the disease.

ITIH4, a key acute-phase protein in the ITI family, has a well-documented role in inflammation [42,43]. However, in RA, only upregulation of ITIH4 expression and its citrullinated form has been reported previously. In this study, we illustrated the mechanistic role of ITIH4 through a detailed in silico study by analyzing the disease-specific interacting protein of ITIH4 and involved signaling pathways, followed by in vitro validation by ITIH4 knockdown in RA-FLS.

Interacting targets of ITIH4 were retrieved by reviewing the literature and using the STRING database. Further comparing these targets with RA-associated proteins, 18 interacting proteins of ITIH4 proteins were found to be linked with RA pathogenesis. To identify the involved signaling pathway regulated by ITIH4, GO, and KEGG and Reactome pathway enrichment analysis was conducted, which majorly depicted the major involvement of the chemokine signaling. Further PPI network construction indicated that CXCR4, CCR5, CCL4, and CXCR5 are associated with chemokine signaling as well as are the first interacting neighbors of ITIH4. This indicated that ITIH4 has a major role in regulating the chemokine signaling associated with RA pathogenesis.

Chemokines are small chemotactic cytokines that regulate immune cell migration, playing a crucial role in maintaining homeostasis, generating immune responses, and contributing to disease pathology. Their primary functions include leukocyte migration, cell proliferation, survival, differentiation, degranulation, and cytokine production. Chemokines and their receptors are essential for trafficking and activating monocytes and lymphocytes at inflammation sites, facilitating the recruitment of circulating leukocytes. They also play a significant role in the development of RA, influencing both inflammation and angiogenesis [44]. Chemokines and chemokine receptors are highly abundant in the peripheral blood, synovial fluids, and in the inflamed joints of RA [45]. Studies have also suggested the direct role of the chemokine system in the destructive phase of RA by modulating the migration, proliferation, and MMP production by RA-Fibroblast-like synoviocytes (RA-FLS) [46]. Some blockades have demonstrated promising results in early-phase clinical trials, indicating that chemokine ligand–receptor interactions continue to be a promising therapeutic target for RA [47].

Further molecular docking analysis of 18 screened proteins with ITIH4 demonstrated the strong binding with major proteins CXCR4, CXCR5, CCR5, and CCL4, which are also found to be involved in chemokine signaling. Among them, interaction with CXCR4 provided the best weightage energy score of −1705.7 kcal/mol.

C-X-C chemokine-receptor type 4 (CXCR4) is a seven-transmembrane, G protein-coupled receptor (GPCR) mainly expressed on leucocytes, endothelial, epithelial, and hematopoietic stem cells, and stromal fibroblasts [40,47]. The downstream signaling of CXCR4 involves the PI3K/AKT, NF-kB, JAK/STAT, and RAS-ERK-MEK pathways, which play roles in cell proliferation, angiogenesis, invasion, division, metastasis, and inflammation [40]. CXCR4 is abundantly expressed in the RA serum and joint synovial fluid [48,49] and the entry of T cells into the synovium is also primarily regulated by CXCR4 [50,51,52]. The CXCL12/CXCR4 axis facilitates monocyte migration into the joints of RA ST-transplanted SCID mice [53] and enhances the production of both MMP-9 and MMP-13 in human chondrocytes [54]. CXCR4/CXCL12 axis also regulates the migration of B cells into RA synovium [55] and showed angiogenic activity in RA synoviocytes [56], auto-antibody production, and arthritic symptoms in a collagen-induced arthritis (CIA) model [57,58]. These indicated that CXCR4 has a major role in RA pathogenesis, and activation of CXCR4, and associated signaling pathways lead to RA progression.

To validate our in silico data depicting the association of ITIH4 with CXCR4, in vitro ITIH4 knockdown studies in RA-FLS were conducted. Significant downregulation of ITIH4 and CXCR4 expression was observed after silencing ITIH4 in RA-FLS using ITIH4 siRNA. This directly proportional relation of ITIH4 and CXCR4 expression in knockdown assay signifies that ITIH4 increased expression triggers CXCR4 activation, suggesting that ITIH4 might act as a ligand for CXCR4 receptor regulating chemokine mediated signaling pathways in RA-FLS.

Therefore, combining our in silico and in vitro observation, we speculated that ITIH4 can promote RA progression via acting as a ligand for the CXCR4 receptor in RA synoviocytes, activating the CXCR4 signaling pathway, and further regulating the disease-promoting molecules such as phosphorylation of PI3/Akt complex, activation of JAK and Src kinases, phosphorylation of p65 complex and their associated pathways PI3/Akt/mTOR pathway, JAK/STAT pathway, Ras/Raf/MEK/ERK, and NF-κB pathway respectively [59]. Therefore, targeting ITIH4-CXCR4 can be a promising therapeutic strategy for managing RA inflammation and progression via suppression of CXCR4-mediated signaling pathways. In the future, in-depth pathway analysis of ITIH4 through targeted in vitro and in vivo validations are necessary to illuminate and validate the role of ITIH4 in RA, discovering a major protein mediator in RA pathogenesis.

## Figures and Tables

**Table 1:** The clinical demographic characteristics of RA patients.

S. No.	Patient’s Characteristics	RA (*n* = 3)
1	Age (yrs.)	50 ± 5
2	Sex (Female, Male)	F (2), M (1)
3	ESR (mm/h)	35 ± 5
4	RF (+ve/−ve)	+ve
5	CRP (mg/L)	80 ±15
6	Tender Joint	20 ± 6
7	Swollen joints	10 ± 4
8	DAS-28 score	6 ± 0.5
9	Disease duration yrs.	10 ± 5
10	Medication (Yes/No)	Yes

**Table 2:** Binding sites of the active site of the ITIH4 protein predicted by using CASTp.

Chain	Binding Sites
A	36TYR, 37SER, 39THR, 41ASP, 42SER, 43ARG, 44VAL, 45SER, 46SER, 47ARG, 48PHE, 50HIS, 52VAL, 54THR, 56ARG, 78PHE, 80THR, 81ASN, 85ILE, 87ASP, 88GLY, 141THR, 143GLU, 145VAL, 147GLU, 149LEU, 150LEU, 151LYS, 152ARG, 153ARG, 154LEU, 155GLY, 169VAL, 170LYS, 171HIS, 173GLN, 174MET, 175ASP, 177HIS, 179PHE, 180GLU, 181PRO, 182GLN, 183GLY, 184ILE, 185SER, 187LEU, 190GLU, 205TRP, 206GLN, 207ASN, 208LYS, 209THR, 210LYS, 211ALA, 212HIS, 214ARG, 215PHE, 216LYS, 217PRO, 218THR, 219LEU, 221GLN, 222GLN, 225SER, 239ILE, 241TYR, 242ASP, 243VAL, 244ASP, 245ARG, 246ALA, 247ILE, 248SER, 249GLY, 250GLY, 251SER, 252ILE, 253GLN, 254ILE, 255GLU, 256ASN, 257GLY, 265PRO, 266GLU, 268LEU, 269THR, 270THR, 271MET, 272PRO, 273LYS, 274ASN, 275VAL, 288ARG, 292GLN, 294ARG, 295GLU, 298ILE, 299LYS, 301LEU, 302ASP, 303ASP, 304LEU, 305SER, 306PRO, 307ARG, 308ASP, 309GLN, 326LEU, 329ALA, 331ALA, 334VAL, 338ARG, 342ALA, 363LEU, 366SER, 367ASN, 370GLU, 371ARG, 372LEU, 373PRO, 374GLU, 375GLY, 376SER, 377VAL, 378SER, 379LEU, 380ILE, 381ILE, 405ALA, 406VAL, 408GLY, 409ARG, 410TYR, 411SER, 413PHE, 431LEU, 432ASP, 433ASN, 434GLY, 435GLY, 436LEU, 438ARG, 439ARG, 440ILE, 441HIS, 442GLU, 443ASP, 444SER, 445ASP, 447ALA, 448LEU, 449GLN, 451GLN, 452ASP, 453PHE, 454TYR, 455GLN, 456GLU, 457VAL, 458ALA, 459ASN, 460PRO, 461LEU, 462LEU, 463THR, 464ALA, 465VAL, 467PHE, 469TYR, 470PRO, 471SER, 472ASN, 473ALA, 476GLU, 478THR, 479GLN, 480ASN, 482PHE, 483ARG, 484LEU, 485LEU, 486PHE, 487LYS, 488GLY, 489SER, 490GLU, 491MET, 492VAL, 493VAL, 494ALA, 495GLY, 496LYS, 497LEU, 498GLN, 500ARG, 502PRO, 504VAL, 505LEU, 506THR, 507ALA, 508THR, 509VAL, 513LEU, 514PRO, 515THR, 516GLN, 517ASN, 518ILE, 519THR, 520PHE, 521GLN, 522THR, 524SER, 525SER, 526VAL, 537LYS, 538TYR, 539ILE, 540PHE, 542ASN, 545GLU, 546ARG, 573ASN, 576LEU, 577ASN, 578LEU, 580LEU, 581ALA, 582TYR, 583SER, 584PHE, 585VAL, 586THR, 587PRO, 588LEU, 590SER, 591MET, 593VAL, 594THR, 595LYS, 596PRO, 597ASP, 599GLN, 600GLU, 601GLN, 604VAL, 615ASN, 617ASN, 618VAL, 621GLY, 624PHE, 625PHE, 627TYR, 628TYR, 629LEU, 630GLN, 631GLY, 632ALA, 633LYS, 634ILE, 635PRO, 636LYS, 647TRP, 649ARG, 650GLN, 651ALA, 652GLY, 653ALA, 654ALA, 655GLY, 656SER, 657ARG, 658MET, 659ASN, 660PHE, 661ARG, 662PRO, 663GLY, 664VAL, 665LEU, 666SER, 667SER, 668ARG, 669GLN, 670LEU, 671GLY, 672LEU, 673PRO, 674GLY, 675PRO, 677ASP, 678VAL, 679PRO, 681HIS, 682ALA, 683ALA, 684TYR, 685HIS, 686PRO, 687PHE, 688ARG, 689ARG, 690LEU, 691ALA, 692ILE, 693LEU, 694PRO, 695ALA, 696SER, 697ALA, 698PRO, 699PRO, 703ASN, 705ASP, 707ALA, 708VAL, 710ARG, 711VAL, 712MET, 714MET, 715LYS, 716ILE, 717GLU, 718GLU, 719THR, 720THR, 721MET, 722THR, 723THR, 724GLN, 725THR, 726PRO, 727ALA, 728PRO, 729ILE, 730GLN, 731ALA, 732PRO, 733SER, 734ALA, 735ILE, 736LEU, 737PRO, 738LEU, 739PRO, 741GLN, 742SER, 744GLU, 745ARG, 746LEU, 749ASP, 750PRO, 751ARG, 754GLN, 755GLY, 756PRO, 757VAL, 758ASN, 759LEU, 760LEU, 761SER, 762ASP, 763PRO, 764GLU, 765GLN, 766GLY, 767VAL, 768GLU, 769VAL, 770THR, 771GLY, 772GLN, 773TYR, 774GLU, 775ARG, 776GLU, 777LYS, 778ALA, 779GLY, 780PHE, 781SER, 782TRP, 783ILE, 784GLU, 785VAL, 786THR, 787PHE, 788LYS, 789ASN, 790PRO, 791LEU, 792VAL, 793TRP, 794VAL, 795HIS, 796ALA, 797SER, 798PRO, 799GLU, 800HIS, 801VAL, 802VAL, 803VAL, 807ARG, 810SER, 811ALA, 812TYR, 814TRP, 815LYS, 820SER, 821VAL, 822MET, 823PRO, 824GLY, 825LEU, 826LYS, 837LEU, 845ILE, 846GLY, 847LEU, 849PHE

**Table 3:** Protein–protein interaction: Weighted score of lowest energy of A chain of ITIH4 protein and chains of 18 common proteins along with number of H-bonds.

S.No.	Ligand Protein–Receptor Protein	Weighted Scores of Lowest Energy (kcal/mol)	No. of H-Bonds
1.	ITIH4-SCD5 (Chain A: Chain A)	−1814.8	None
2.	ITIH4-CXCR5 (Chain A: Chain A)	−1733.3	None
3.	ITIH4-CXCR4 (Chain A: Chain B)	−1705.7	6 (A: B)
4.	ITIH4-TG (Chain A: Chain B)	−1514.8	19 (A:B)
5.	ITIH4-CCR5 (Chain A: Chain B)	−1440.6	5 (A: B)
6.	ITIH4-SDC3 (Chain A: Chain A)	−1300.6	None
7.	ITIH4-FN1 (Chain A: Chain A)	−1271	None
8.	ITIH4-CD209	−1184.9	20 (A: B)
9.	ITIH4-ACTG1 (Chain A: Chain B)	−1148	10 (A: B)
10.	ITIH4-CD8A (Chain A: Chain A)	−1139.7	None
11.	ITIH4-ALB (Chain A: Chain B)	−1008.6	10 (A: B)
12.	ITIH4-ACTBL2 (Chain A: Chain A)	−980.9	None
13.	ITIH4-PGK1 (Chain A: Chain B)	−968.6	16 (A: B)
14.	ITIH4-CCL4 (Chain A: Chain B)	−934.6	23 (A: B)
15.	ITIH4-HSPA1B (Chain A: Chain A)	−933	None
16.	ITIH4-CR2 (Chain A: Chain B)	−873.9	8 (A: B)
17.	ITIH4-CD4 (Chain A: Chain B)	−863.3	19 (A: B)
18.	ITIH4-VTN (Chain A: Chain B)	−855.1	11 (A: B)

**Table 4:** H-bond length between amino acid residues of chain A of ITIH4 and chain B of CXCR4.

AA Residues of Chain A ITIH4	AA Residues of Chain B CXCR4	Bond Length (Å)
HIS 441	ASN 101	3.05
ARG 288	ASP 182	2.74
ASP 443	TYR 184	2.87
SER 927	LYS 239	2.69
SER 927	LYS 239	2.70
LYS 831	GLN 314	2.44

## Data Availability

Data are contained within the article or Supplementary Materials.

## Supplementary Materials

The following supporting information can be downloaded at https://www.mdpi.com/article/10.3390/pathophysiology31030038/s1. File S1: Gene ontology and pathway enrichment analysis of common targets.
